# Realization times of energetic modernization measures for buildings based on interviews with craftworkers

**DOI:** 10.1038/s41597-023-02379-6

**Published:** 2023-07-20

**Authors:** Jan Richarz, Nico Fuchs, Jacqueline Zurke, Jan Imberg, Tanja Datsko, Dominik Hering, Dirk Müller

**Affiliations:** RWTH Aachen University, E.ON Energy Research Center, Institute for Energy Efficient Buildings and Indoor Climate (EBC), Aachen, Germany

**Keywords:** Civil engineering, Mechanical engineering

## Abstract

Modernizing existing buildings is vital to saving carbon emissions and counteracting global climate change. Many countries face the challenge of modernizing a considerable number of their buildings in the next two decades. Implementing related modernization measures requires a high number of craftworkers. However, current studies indicate that these craftworkers’ current lack will increase. Examining the effects of craftworkers shortage on modernization strategies needs data concerning the realization time of modernization measures. We collected this data based on 90 expert interviews and provide it in this paper. The interview results comprise realization times for insulation measures of the envelope and changes in the heat supply system for typical buildings. This paper describes the data collection and presents the raw data available at a repository at Figshare. The data is internationally applicable in simulation and optimization approaches for building modernization.

## Background & Summary

Existing buildings cause 28% of global carbon emissions^1^. More than 50% of European buildings were built before 1980^2,3^ without ambitious regularities on energy efficiency. Since these buildings are responsible for more than 60% of all European buildings’ heating and cooling demand^4^, a significant emission-saving potential lies in modernizing them and the majority of the building stock.

The targeted emission savings through the modernization of buildings require diverse resources such as the availability of craftworkers, low-emission heating devices, and insulation material. These resources are only available in limited quantities, which should be considered during realistic long-term modernization planning. Among the limited resources is the capacity of the craftwork to realize modernization measures. For example, Seefeldt *et al*.^5^ determine that up to 130,000 additional workers are needed for the entire energy transition in Germany, of which the heating sector will account for about 20,000 by 2025. The study concludes that the skilled labor gap in this sector will worsen by 2035^5^. Kenkmann *et al*.^6^ assume a similar labor gap and recommend a priority role for securing skilled labor in the energy transition context. Amann *et al*.^7^ identify the lack of suitable particularly skilled workers, as a critical barrier to expanding the modernization rate. Schirner *et al*.^8^ show that in 2020, among the 13 skilled trades categories with the most significant shortages of skilled workers in Germany, four categories belong to construction, architecture, and surveying and building services engineering. The occupational category plumbing, heating, and air-conditioning technology had the second largest shortage of skilled workers, with over 10,000 vacancies^8^. Münch *et al*.^9^ examine labor potentials in the building sector and conclude that the lack of skilled workers in the building sector poses a risk for the energy system transformation. Juricic *et al*.^10^ review the construction labor demand and shortages in the European Union and state that most member states face labor and skill shortages in various sectors, including the construction sector. The studies mentioned highlight that the capacity of craftworkers is likely to be a bottleneck for the energy transition in the building sector.

Craftwork capacities should be included in modernization strategies to consider the effects of the shortage of skilled workers. For detailed analyses of the influences of limited craftwork capacities for building modernizations, data about the time needed to realize modernization measures by craftworkers is necessary. This data could be integrated into various research approaches: Ranging from scenario analyses of the whole building energy transition to long-term building modernization models of single buildings and building portfolios.

In scenario analyses of the building energy transition, as in the work of Luderer *et al*.^11^ or the German Energy Agency^12^, the authors examine different pathways to climate neutrality in the German building stock. In these top-down models, craftwork realization times could be set as a an additional limitation to investigate the diffusion of technologies. Moreover, also recent works for the long-term planning of modernization measures in single buildings could benefit from the data on craftwork realization times: The works of Petkov *et al*.^13^ and Richarz *et al*.^14^ show developments of modernization roadmaps where influences of limited craftwork capacities on modernization decisions in single buildings could be analyzed. Data regarding the realization times of modernization measures could also help in the modernization planning of building portfolios, for example, in the following studies: In the work of Pannier *et al*.^15^, where the authors identify optimal renovation schedules for a building portfolio of multi-family houses by simulating different modernization measures and choosing the optimal combination with a genetic algorithm. Furthermore, Fernandez-Luzuriaga *et al*.^16^, identify cost-optimal levels for the energy refurbishment of a residential building stock scaled up from optimal results on single building level.

Integrating craftwork capacities into these research approaches make it possible to investigate how to achieve climate goals at different levels (e.g., single buildings, building stock) despite limited craftwork capacities and how technology decisions change within this context. Furthermore, building planners and architects in practice can use this data in their early-stage modernization concepts.

Existing data on modernization measures’ realization time is partly available for calculating specific measures but not for modernizing typical buildings. However, the analyses of typical buildings can provide general evidence. Construction estimators often use internal company data for their calculations. Commercial databases for realization times, such as Sirados^17^ and Bürgerle^18^, exist. Still, they do not include holistic data for all relevant modernization measures, are limited to specific example measures, are challenging to use, and are not freely available. Existing studies regarding realization times (e.g. Kloep^19^) only include data for individual construction areas, often do not focus on modernization measures, and only publish excerpts of aggregated data.

Thus, to our knowledge, available data for realization times for energetic modernization measures of existing buildings is missing. To address this research gap, we conducted 90 expert interviews with craftworkers of different fields and with different working experience. These craftworkers work in different company locations in Germany and the companies are of different company sizes. Within these interviews, data for realization times for energetic modernization measures of existing buildings was collected. In this paper, we present the results of those interviews.

The paper is structured as follows: Methods describes the examined modernization measures, the types of interviews, and the interview guidelines. In Data Records, we explain the raw data. Technical Validation shows a comparison of the interview results to available literature values. In the Usage Notes, we describe how the data can be used and which influences need to be taken into account. Furthermore, an usage example is presented.

## Methods

### Examined modernization measures

Two categories of measures are typically considered during energetic building modernizations: Improving the building envelope by insulating or changing its components and changing the energy supply system.

The usual measures that can improve the building’s envelope are examined because they can minimize heat loss and improve the thermal comfort of the building. Outer walls, roof (whether flat or pitched), and building ground can be insulated against the air and the ground. Additionally, replacing old windows with energy-efficient ones can be crucial. Double or triple-glazing and insulated frames can significantly reduce ambient heat transfer.

Furthermore, we examined the change in the building’s energy supply system, because it can increase energy efficiency and make it possible to integrate renewable energies which are the key goals of the energy transition. Our focus is on the most promising technologies for single buildings: Existing gas or oil boilers, which typically supply heat to existing European buildings, can be replaced with more efficient and ecological options. For example, condensing gas boilers recover heat from flue gases, while biomass (biom.) boilers utilize renewable organic materials for heating. Combined heat and power (CHP) systems generate electricity and capture waste heat for space heating or hot water. Fuel cells (FC) also provide electricity and heat through electrochemical processes. District heating networks (DH) distribute heat from a central source to multiple buildings, reducing emissions depending on the energy sources of the network. Buildings to be modernized can be connected to existing networks.

Heat pumps (HP) play a crucial role in the energy transition, utilizing electricity and environmental heat for building heating. Air-to-water heat pumps (AtWHP) use surrounding air to heat water, while air-to-air heat pumps (AtAHP) supply tempered air to a building. Ground-source heat pumps (GSHP) extract heat from the ground and therefore require a connection to geothermal collectors (col.) or probes, offering a renewable energy source. In older buildings, radiators (rad.) can be upgraded to increase the heat pump’s efficiency. Many heat pumps also provide both heating and cooling capabilities. For cooling needs, compression chillers (VCRS) can also be employed. These chillers work similarly to heat pumps.

Energy storages play a vital role in the energy transition. Therefore, we considered batteries (BAT) as electrical energy storage and water storages as thermal energy storages (TES). Further, we examined realization times for solar thermal collectors (STC) that utilize the sun’s power to heat water and photovoltaic modules (PV) that directly convert sunlight into electricity. These measures collectively contribute to energy efficiency and sustainable building practices.

### Interview conduction

To collect data for the realization times of the mentioned energetic modernization measures, we conducted 90 expert interviews with craftworkers of different fields and generated the data described in the following. We followed the recommendations of Misoch^20^ for conducting these interviews.

In the first step, we developed a questionnaire that also served as an interview guideline to ensure that every interview is conducted in the same way and quality regardless of who performs it. The questionnaire is added to the repository at Figshare^21^, enabling others to conduct comparable interviews.

This study is based on two types of interviews: On the one hand, personal telephone interviews and on the other hand, a questionnaire, based on the interview guideline that was briefly explained via telephone and then filled out independently. Both procedures consisted of an information phase and a data phase.

In the information phase, the background of the research, personal and company data privacy was explained, and the request for agreement to be allowed to use the data on craftwork realization times and to publish the data in an aggregated and anonymous form. We asked the experts for person-hourly data and not about the duration of the measures. Furthermore, the data shall be average data for typical existing buildings and include all installation steps up to the turnkey measure. We assumed the whole component area would be insulated for insulation measures on the building shell. To delimit the realization times inquired, we noted that waiting times for equipment, delays in deliveries or the construction process, and additional time due to errors in other craftwork fields should not be considered.

In the data phase, the actual data was collected. This part was divided into the realization time for modernization measures concerning the building envelope, the uninstallation of existing devices, and the installation of new devices. If interviewees reported significant influencing factors (e.g., the capacity of existing boilers), values were collected depending on the factors mentioned. Furthermore, some interviewees answered with a range of values for realization times. In these cases, we collected minimum, average, and maximum values.

## Data Records

All collected data from the interviews are available at a repository located at Figshare^21^. The repository comprises a xlsx-file, a json-file, and a folder with several .csv-files containing the raw data for realization times, and the questionnaire. The xlsx-file is organized as follows: The first sheet provides an overview of the data in the file. The second sheet shows the mean values and 95% confidence intervals for the realization times of each modernization measure. For each modernization measure, a separate sheet displays the collected data. The first row of each sheet contains the name of the modernized component, while the second row describes the data listed along with its respective unit. The following rows present the data, with each interview data on a single line. Each interview is assigned an interviewer ID and a company ID to ensure data confidentiality. The date column indicates the interview date, while the measure column identifies the corresponding modernization measure. Depending on the specific modernization measure, data on minimum, average, and maximum values for small or large devices are available. For every measure, at least average values are provided.

The .json and the csv-files offer the same raw data as the xlsx-file. The structure of the .json-files is similar to the structure of the xlsx-files. On the highest level, one object presents the mean values, while the others present different modernization measures. Each object consists of the name of the data set, the content of the sheet, a short description, and a further data object including one object for each interview result. The data object for the mean values distinguishes the different modernization measures and gives the measure’s mean values and 95% confidence intervals. The data objects for the various modernization measures present information about the interviewer, the interviewee, and the collected data of the respective interview.

The csv-files directly follow the structure of the xlsx-file presented in different files for each sheet of the xlsx-file. Besides the raw data, the questionnaire, i.e., interview guideline used in the expert interviews, are presented in the repository. Other data can be collected by using this guideline.

## Technical Validation

To validate the interview data, we compare the realization times of the interviews with literature values. This section uses data from Plümecke^22^, Sirados^17^, ARH^23^, Kloep^19^, and Bürgerle^18^.

Plümecke^22^ gives calculation aids for insulation measures with thermal composite systems. Sirados^17^ is an online database representing various construction measures’ costs and realization times from lists of actual construction projects. The database lists sub-tasks of modernization measures that can be combined to complete modernization measures. Since this database reflects the duration of the construction work, the time data must be further processed to calculate person-hours using the number of craftworkers required. The interviewees told us that two workers typically work in parallel for measures on the building envelope and the energy supply system.

ARH^23^ gives guideline values for the insulation of outer walls. Kloep^19^ shows realization times for installing air-to-water heat pumps in existing buildings derived from interviews. Bürgerle^18^ is a calculation software for the realization times of technical building measures. The validation is limited because the diverse literature sources consider a different scope of sub-measures and, for example, do not consider partially preparatory work or traveling time. In some cases, the scope of the sub-measures under consideration is not apparent.

**Fig. 1:**
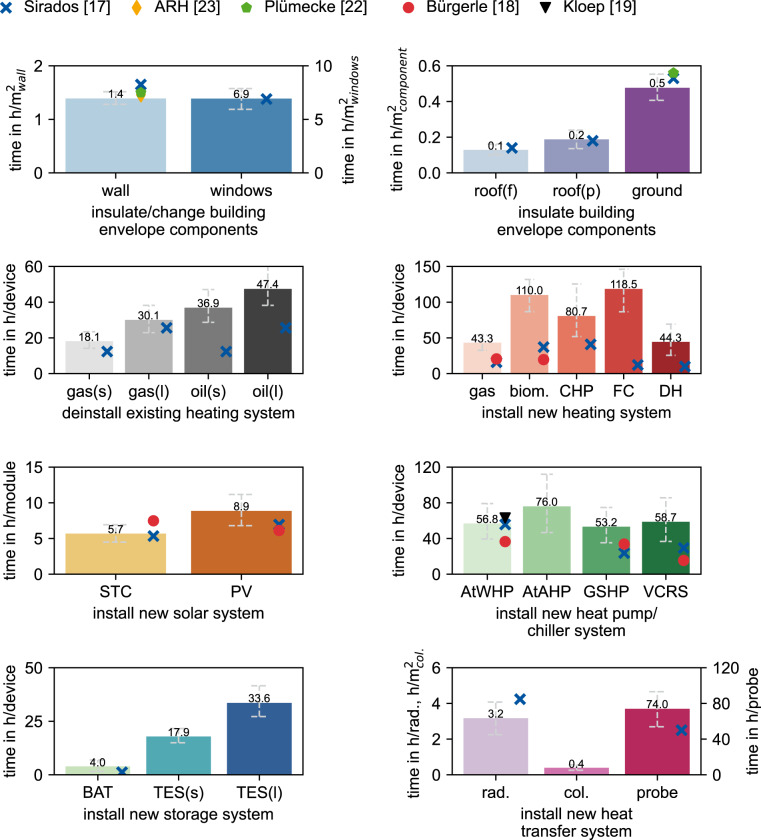
Average data and error bars of the generated data for the realization time of modernization measures by craftworkers based on 90 expert interviews compared to literature values (where available). Abbreviations: flat (f), pitched (p), small (s), large (l), biomass (biom.), combined heat and power (CHP), fuel cell (FC), district heating (DH), solar thermal module (STC), photovoltaics modul (PV), air-to-water heat pump (AtWHP), air-to-air heat pump (AtAHP), ground-source heat pump (GSHP), vapour-compression refrigeration system (VCRS), battery (BAT), thermal energy storage (TES), radiator (rad.), geothermal collector (col.), geothermal probes (probe).

However, Fig. 1 compares interview and literature data for all modernization measures queried in the interviews. For the interview data, average values are shown in bars and 95%-confidence intervals determined by bootstrapping are indicated in grey error bars.

Realization times for modernization measures on the building envelope greatly agree with literature values. For the insulation of the outer wall, data from interviews is similar to the value of Plümecke^22^ for composite thermal insulation, while the values of Sirados^17^ and ARH^23^ deviate less than 7%.

Regarding the uninstallation of existing boilers, the data of Sirados^17^ for gas boilers are 15–32% lower and the data for oil boilers are 46–67% lower than the interview data. Deviations can be explained by the fact that the database does not include the planning of the measures and transport times. It further does not distinguish the efforts for oil and gas boilers, suggesting that the uninstallation of oil storages is not included in the database.

Data for installing new heating systems from the literature are lower than the interview data. The differences presumably result from the fact that planning and transport were not considered. Different amounts may have been calculated for preparatory measures in the construction process, or ideal conditions were assumed. In contrast, we asked for typical values in the interviews. Regarding the realization times from Sirados^17^, it must be mentioned that implementing storage systems is also a part of the realization time for implementing heat pumps and pellet boilers and cannot be separated. This additional storage implementation leads to minor deviations in realization times compared to interview data.

Regarding the installation of air-to-water heat pumps, interview data show good agreement with the data of Kloep^19^, which also considers the realization times up to the turnkey measure.

For solar thermal collectors, data from Sirados^17^ show good accordance with the interview data, while Bürgerle^18^ gives a 30% higher value. For photovoltaic systems, literature data from Sirados^17^ and Bürgerle^18^ lies below the interview data. The exact reasons for the deviations in installing of heating systems can be suspected here.

Realization times from Sirados^17^ for batteries are lower than the interview data. For thermal storage systems, no data from the literature is available. Regarding the exchange of radiators, realization times from Sirados^17^ are slightly higher than the interview data. In contrast, data for geothermal probes lies in the lower range of confidence intervals from the interviews.

The comparison shows good accordance for modernization measures on the building envelope, while realization times of measures on the heating and solar systems exceed literature values. This exceedance can be justified because the referenced calculation aids only consider the installation work on the construction site and not the time for planning and transport, which the interviews do. Data from Sirados^17^ does not explain the number of workers needed, which could lead to person-hours deviations if employees work in parallel.

Based on the interviews, we assume that planning and transport tie up an essential part of the capacities of craftworkers. Considering or not considering these factors could explain the differences between interview data and the literature sources.

## Usage Notes

In this chapter, some advice for data usage is given, the representativity of the sample is discussed, and a published usage example of the data is presented.

### Usage advice

Some qualitative information and influences on the data were provided during the interviews. Since these influences could be necessary when using the data, they are described in the following. Concerning envelope insulation and the change of windows, the experts provided area-specific data. According to the experts, these values are transferable to many other buildings but vary for complex building cubatures or other exceptional cases. Conversely, the installation time of new heating devices in typical buildings does not scale by size but by piece. For solar-powered systems, module-specific values are provided, and experts stated that the surrounding infrastructure (e.g., cabling) increases linearly with the number of modules. Installation times for probes are provided per piece for typical depths, while values for collectors are provided per collector area.

The presented results comprise typical values that can be utilized to estimate the realization times of modernization measures in typical buildings. However, the interviewees told us some factors that could result in deviations from these typical values when applied to individual buildings. Table 1 summarizes these influences on realization times and categorizes them into general factors, factors affecting the building envelope, and the energy supply system.

**Table 1:** Key influences on the effort of modernization measures.

Category	Measure	Influencing factors
General	—	Qualification of workers
	—	Transport distances
	—	Accessibility of the workplace
	—	Coordination with other construction services
	—	Weather and environmental factors
Building envelope	External wall insulation	Type of insulation and materials
		Building geometry and condition
		Architectural requirements
	Roof	Type of construction and structure
		Roof condition and existing materials
	Ground Floor	Structure and layout of floor/ceiling construction
		Installation method
	Window replacement	Window properties
		Type and condition of envelope
Energy supply	Heat conversion units	Existing hydraulic system
		Installation conditions
	Heat transfer system	Layout of the rooms
	Solar systems	Roof structure
		Integration into BES
	Geothermal energy	Soil properties

As a general factor, worker qualification influences their productivity, affecting the realization time of modernization measures. The availability of qualified workers varies regionally and depends on the labor market situation. Furthermore, transport distances influence the realization time, with longer transport distances resulting in more travel time and hence more time for implementing measures. The accessibility of the workspace differs under specific building boundary conditions, especially for scaffolding and geothermic, and significantly affects preparatory and follow-up work durations. The coordination with other construction services may also lead to deviations in the realization times, as synergies can accelerate implementation, while conflicts can hinder it. The weather and environmental factors impact worker productivity and construction progress for measures carried out outside the building (external insulation, implementation of solar systems).

Regarding modernization measures on the building envelope, building geometry, condition, type of insulation and materials, construction types, and architectural requirements (e.g., historical preservation) affect the realization time. A complex building geometry requires additional effort, and bad surface conditions require more preparatory work before installing the insulation. Further, the structure and layout of the envelope components of the modernized building influence realization times as they decide about the installation method of the insulation. Complex layouts may lead to further efforts, while simple structures may fasten realization times.

Concerning modernization measures of the energy supply system, the existing hydraulic system influences the required extent of modernizing heat supply devices. Additionally, the effort is influenced by the layout and accessibility of the utility room, with complex geometries leading to additional effort. This applies especially to uninstalling existing gas or oil boilers which must be dismantled inside the building before transport above a specific device size. Considering solar systems, the necessity of preparation measures depends on the structure of the roof and existing cables, influencing realization times. Regarding the realization times of geothermal probes, the soil properties affect the efforts required for drilling borehole heat exchangers.

We do not estimate significant country-specific influences in the data and assume it could be used internationally.

### Representativity of the sample

The data is based on a sample of 90 interviews with craftworkers, which provided valuable insights into the needed time for the realization of modernization measures of buildings and provided data that has not been available so far. Three aspects were considered to achieve a sufficient representativity of the data set: Criteria for selecting the experts, i.e., interviewees, the triangulation of the data, and the saturation of new insights.

Concerning selection criteria, we aimed to generate a diverse sample by choosing craftworkers of different working experience, different company sizes, from different regions, and specializations regarding different modernization measures in different building types. Two different interview methods were chosen to complete the data triangulation: Personal telephone interviews with an interview guideline and an independent questionnaire based on the guideline. The convergence of data from the two sources enhances the robustness and generalizability of the data. Finally, we recognized a saturation of new insights with an increasing number of conducted interviews. For most of the data, craftworkers gave similar values and named the same influences on these values. This can, for example, be seen in the data for conventional devices like gas boilers with low deviations. Higher deviations represent newer devices since different experiences with these technologies exist under the craftworkers. The experts also confirmed these different experiences and are present within and across companies.

However, a larger sample may enhance the statistical power and generalizability of the data, which could be an issue for further research using this paper’s method and interview guideline. Since our interviews lasted between one and two hours each, it is essential to consider the context and feasibility of conducting such interviews within the possible resources of the research.

### Usage example

Different data applications are possible: For research purposes, the data can be used in model-based approaches for long-term modernization strategies to determine the effects of limited craftwork capacities. This usage is possible at various levels of detail: From individual building considerations to urban energy systems and building portfolios up to studies of building stocks.

A usage example to consider the data in long-term modernization measures of a single building is described in Richarz *et al*.^24^. In that work, a method to handle limited material and immaterial resources in optimization models has been developed. The interview data of this paper was used as an application of this method. The optimization model decides the optimal modernization measures for a single building. Therefore, a calculation of the necessary time to realize all measures selected by the model was implemented. Next, this time can be constrained, and the model’s decision change accordingly. The effects of limited craft capacities can be derived by analyzing different levels of available craftwork realization time. The results show that the limitation of craftwork capacities significantly changes the optimal modernization decisions. Policymakers could use the results to incentivize the training of craftworkers in key modernization measures to reduce the corresponding realization times.

The data is also applicable for the early stage planning of modernization measures and the controlling during a construction project.

## Data Availability

No costum code was used for the method.
